# Hyperammonemic Encephalopathy and Thyroid Storm Leading to Coma: A Case Report

**DOI:** 10.1155/crcc/2330763

**Published:** 2025-07-03

**Authors:** Tung Phi Nguyen, Thang Trong Khong, Tuan Duc Tran

**Affiliations:** ^1^Intensive Care Unit, Vinmec Central Park International Hospital, Ho Chi Minh City, Vietnam; ^2^Diagnostic Imaging Department, Vinmec Central Park International Hospital, Ho Chi Minh City, Vietnam

**Keywords:** coma, critical care, hyperammonemic encephalopathy, non-hepatic hyperammonemia, thyroid storm

## Abstract

Hyperammonemic encephalopathy is a rare but serious condition often linked to hepatic dysfunction or metabolic disorders. Thyroid storm is an endocrine emergency that can cause profound metabolic disturbances, though its relationship with hyperammonemia remains unclear. We report a unique case of a 33-year-old previously healthy male who developed progressive fatigue, nausea, and vomiting, followed by acute neurological deterioration. Initial evaluation revealed hyperammonemia (284 *μ*mol/L) and thyrotoxicosis (fT4: 27 pmol/L and TSH: 0.007 *μ*IU/mL), with no hepatic dysfunction. His neurological status declined from GCS 12 to GCS 7 within hours. Brain MRI and cerebrospinal fluid analysis were unremarkable. The patient was diagnosed with coma in the setting of hyperammonemic encephalopathy and thyroid storm. Management included propylthiouracil, Lugol's iodine, propranolol, hydrocortisone, and continuous renal replacement therapy (CRRT). His ammonia levels significantly improved within 48 h, leading to rapid clinical improvement and extubation. This case highlights the importance of recognizing hyperammonemia as a potential cause of altered mental status, even in the absence of hepatic dysfunction, and raises questions about potential but unestablished links between thyroid dysfunction and ammonia metabolism.

## 1. Introduction

Acute alterations in the consciousness require prompt evaluation and identification of the underlying cause. Hyperammonemic encephalopathy (HE) is typically associated with hepatic dysfunction, metabolic disorders, or urea cycle abnormalities [1, 2]. Thyroid storm is an endocrine emergency characterized by severe thyrotoxicosis and multisystem dysfunction. Although both conditions are well-documented causes of altered mental status [1–4], their concurrent occurrence without a clearly established causal relationship is exceptionally rare and warrants further investigation [5, 6].

This case report describes a previously healthy young adult who developed coma in the setting of severe hyperammonemia and thyroid storm. The absence of hepatic disease and other known causes of hyperammonemia posed a diagnostic challenge. While hyperthyroidism is not traditionally linked to ammonia metabolism, this case raises the possibility of unexplored metabolic interactions. By reporting this case, we aim to contribute to the growing body of literature on hyperammonemia in nonhepatic patients and highlight the need for further investigation into potential endocrine–metabolic interactions.

## 2. Case Presentation

A 33-year-old male with no prior medical history, no known liver disease, and no history of alcohol use, drug abuse, or prescription medication use, including valproic acid or carbamazepine, was in his usual state of health until 3 days before admission when he experienced dizziness, nausea, vomiting, and fatigue. He sought medical attention and was prescribed acetylleucine, betahistine, and metoclopramide, but his symptoms persisted. On the day of admission, his family found him unresponsive and brought him to the emergency department.

**Figure 1:**
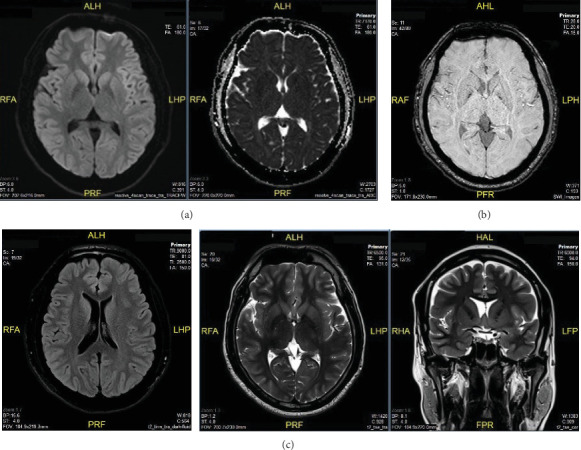
Brain MRI findings. (a) DWI-ADC sequences show no ischemic stroke. (b) SWI sequence shows no intracranial hemorrhage. (c) FLAIR/T2W sequences show no characteristic findings of hyperammonemic encephalopathy.

On arrival, he was stuporous with a Glasgow Coma Scale (GCS) score of 12 (E4V3M5). His pupils were equal and reactive (2 mm), heart rate was 130 bpm, blood pressure was 120/70 mmHg without vasopressor support, and SpO_2_ was 96% on room air. There was no fever, seizures, or meningeal signs. Arterial blood gas obtained on admission revealed a pH of 7.43, pCO_₂_ of 34.6 mmHg, HCO_₃−_ of 23.1 mmol/L, and base excess (BE) of − 1 mmol/L, consistent with mild respiratory alkalosis. Initial laboratory tests showed blood glucose of 8 mmol/L; serum electrolytes are within normal ranges (sodium 146.4 mmol/L, potassium 3.73 mmol/L, chloride 111.2 mmol/L, and calcium 2.40 mmol/L); creatinine 84.4 *μ*mol/L; eGFR 104.15 mL/min/1.73 m^2^; normal liver function tests (AST 15.4 U/L, total bilirubin 25.09 *μ*mol/L, albumin 44.07 g/L, and total protein 70.38 g/L); INR 1.0; and CRP 0.57 mg/L. Serum ketones were negative, urine ketones were mildly positive, and serum lactate was 1.66 mmol/L. The ammonia level on presentation was 213 *μ*mol/L (normal 18–72 *μ*mol/L). Venous blood was collected in EDTA tubes, kept cold, and processed within 30 min using cold centrifugation. Analysis was performed using a Roche Cobas c501 analyzer with an enzymatic UV method. Brain MRI findings showed no ischemic stroke, intracranial hemorrhage, or characteristic findings of HE (Figure 1).

His neurological status deteriorated to GCS 7 (E1V1M5), 3 h after admission. A second ammonia sample, drawn from both central and peripheral venous access, showed a further increase to 284 *μ*mol/L. Cerebrospinal fluid (CSF) analysis revealed normal findings, including a cell count of 7 cells/*μ*L, normal CSF glucose, a normal CSF/blood glucose ratio, and normal protein levels. Autoimmune encephalitis markers were also negative, further ruling out infectious or autoimmune etiologies. Thyroid function tests revealed significant thyrotoxicosis with fT4: 24.6 pmol/L (normal 8–14 pmol/L); fT3: 4.2 pmol/L (normal 2.5–3.9 pmol/L); and suppressed TSH (0.007 *μ*IU/mL) (normal 0.38–5.33 *μ*IU/mL). Given the rapid neurological deterioration and laboratory findings, the patient was intubated, admitted to the intensive care unit (ICU), and started on propylthiouracil, Lugol's iodine, propranolol, and hydrocortisone for thyroid storm. Continuous renal replacement therapy (CRRT) was initiated to manage severe hyperammonemia, and parenteral nutrition with 10% glucose and intravenous lipid was started. Additionally, due to cloudy urine with proteinuria, a urine culture was performed, and empirical antibiotic therapy with ertapenem was initiated. Details of the treatment course and the patient's clinical response over time are presented in Table 1.

At 24 h after admission, he became responsive to verbal stimuli (GCS 10, E3V1M6), his heart rate decreased to 80 bpm, ammonia levels improved to 41.3 *μ*mol/L, and fT4 remained elevated at 27.4 pmol/L. CRRT was discontinued, and protein was reintroduced into his nutrition. By 48 h, he was fully conscious (GCS 15), with stable hemodynamics and ammonia levels at 46.8 *μ*mol/L. Proteinuria was no longer detected at this time. He was then transferred to the endocrinology ward for further management. Antibiotics were discontinued after 5 days when urine cultures returned negative.

The patient was discharged on hospital Day 6, continuing methimazole for hyperthyroidism. At the 1-month follow-up, he was euthyroid, with no neurological deficits and no recurrence of hyperammonemia.

## 3. Discussion

This case is notable for the rapid onset of coma with complete neurological recovery within 48 h following targeted treatment. The two primary suspected causes of altered mental status in this patient were severe hyperammonemia and thyroid storm. One of the unique aspects of this case is that hyperammonemia without hepatic dysfunction is rarely reported in Asian populations [7, 8], and there is no established causal relationship between hyperammonemia and thyrotoxicosis [5, 6].

The diagnosis of thyroid storm in this patient was well supported by laboratory findings and met criteria under both the Burch–Wartofsky Point Scale (BWPS) with a score of 65 [9] and the Japan Thyroid Association (JTA) classification, fulfilling TS1 (definite thyroid storm) criteria [10]. The patient had thyrotoxicosis along with central nervous system dysfunction (coma, GCS 7), tachycardia (130 bpm), and gastrointestinal symptoms (nausea and vomiting), satisfying the required combination for TS1 [10].

Hyperammonemia was confirmed, as ammonia levels exceeded the diagnostic threshold of ≥ 50 *μ*mol/L [1, 7]. According to guidelines from the pediatric continuous renal replacement therapy (PCRRT) workgroup and the Middle East Hyperammonemia and Urea Cycle Disorders Scientific Group (MHUSG), HE is suspected when acute neurological symptoms (e.g., vomiting, behavioral disturbances, altered consciousness, and coma) or chronic neurological manifestations (e.g., developmental delay and psychiatric or behavioral disorders) are present alongside elevated ammonia levels [1, 7]. Several known mechanisms of hyperammonemia in nonhepatic patients were evaluated. Causes related to increased ammonia production, such as hematologic malignancies or hypercatabolic states, were not present [8, 11]. Impaired ammonia clearance due to portosystemic shunting, urea cycle disorders (UCDs), or medication effects was also not identified [8, 11]. The possibility of a urinary tract infection caused by urease-producing bacteria (e.g., *Proteus*, *Klebsiella*, and *Morganella*) [8, 11] was considered, but urine cultures were negative. Nonetheless, given the presence of proteinuria and hematuria, this etiology could not be completely ruled out. UCDs are rare and scarcely described in Asian populations [7]. Mild respiratory alkalosis on admission, which can be seen in UCDs, is not specific. Plasma amino acid and genetic testing were not performed. The absence of family history (e.g., unexplained neonatal deaths) and the patient's prior unremarkable health status make an inborn metabolic disorder less likely, though a late-onset presentation cannot be excluded. The MHUSG recommends immediate dialysis initiation in adults when ammonia levels exceed 200 *μ*mol/L [1]. Similarly, the European expert group suggests dialysis should be performed when there is rapid neurological deterioration and ammonia > 150–200 *μ*mol/L [8]. Hemodialysis (HD) or CRRT can be used, depending on patient condition and institutional capability [1, 8]. In our center, CRRT is preferred for intubated patients, those with hemodynamic instability, or those at risk for cerebral edema, and it is performed at the bedside.

Determining the exact cause of coma in this patient is challenging, as both hyperammonemia and thyroid storm have been associated with impaired consciousness. Hyperammonemia has a well-established link with neurological dysfunction through its effects on astrocyte metabolism, leading to glutamine accumulation, cerebral edema, glutamate dysregulation, and blood–brain barrier disruption [9, 11]. In contrast, the pathophysiological link between thyroid storm and coma remains less clearly defined, with proposed mechanisms including excessive beta-adrenergic stimulation causing central nervous system stress, altered thyroid hormone metabolism affecting brain function, and direct effects of T4 or intermediary factors such as catecholamines, cytokines, and autoimmunity [10, 12]. Coma is an uncommon manifestation of thyroid storm, with large studies estimating an incidence of 7.4%–8.5% [12]. Additionally, metabolic coma typically presents with nonspecific MRI findings; in hyperammonemia, symmetrical involvement of the cingulate gyrus and insular cortex has been observed [11], while thyroid storm is often associated with normal imaging [12]. CSF findings are also typically unremarkable in both conditions. Notably, reports suggest that coma associated with thyroid storm often presents with markedly elevated fT4 levels, typically 3–5 times the upper normal limit [12], whereas in this case, fT4 was only twice the normal value. Given these considerations, it is impossible to definitively attribute the coma to either hyperammonemia or thyroid storm alone; rather, it is likely that both conditions contributed to the patient's neurological deterioration. The patient's full recovery after targeted management of both conditions further supports the likelihood of a multifactorial etiology.

The relationship between thyroid dysfunction and ammonia metabolism remains unclear [5, 6]. In the literature, hypothyroidism has been associated with hyperammonemia, possibly due to decreased urea synthesis, reduced glutamine synthase activity in the liver, and decreased intestinal motility leading to increased ammonia production. However, no clear association between hyperthyroidism and hyperammonemia has been established. To our knowledge, no definitive pathophysiologic link has been reported between these conditions.

Given the rarity of hyperammonemia in the absence of liver dysfunction, especially in an Asian patient, and the concurrent thyroid storm, this case provides valuable clinical insight. While causality remains unclear, reporting such cases is essential for understanding potential interactions between metabolic and endocrine dysfunctions. Future studies and case series may help elucidate possible underlying mechanisms linking these conditions.

## Figures and Tables

**Table 1:** Summary of treatment course and clinical response.

**Timepoint**	**Clinical status**	**Laboratory findings**	**Management**
Admission	Altered consciousness, GCS 12 (E4V3M5), HR 130 bpm	fT3–fT4–TSH: NA^a^ NH_₃_: 213 *μ*mol/L Brain MRI: normal	Investigation of causes of altered consciousness

3 h after admission	GCS 7 (E1V1tM5), HR 130 bpm, proteinuria	fT4: 24.6 pmol/L fT3: 4.2 pmol/L TSH: 0.007 *μ*IU/mL NH_₃_: 284 *μ*mol/L	Mechanical ventilation, PTU, Propranolol, Lugol's iodine Hydrocortisone, CRRT Parenteral nutrition (glucose + lipids) Antibiotics

24 h after admission	Responsive to verbal stimuli, GCS 10 (E3V1tM6), HR 80 bpm	fT4: 27.4 pmol/L fT3: 3.0 pmol/L TSH: 0.009 *μ*IU/mL NH_₃_: 41.3 *μ*mol/L	Extubation; discontinuation of CRRT Reintroduction of protein in nutrition Continuation of prior treatment^b^

48 h after admission	Fully conscious, GCS 15, HR 70–80 bpm	fT4: 18.8 pmol/L fT3: 2.6 pmol/L NH_₃_: 46.8 *μ*mol/L	Transfer to Endocrinology for further management Continuation of prior treatment^b^

Day 6 of hospitalization	Fully recovered, HR 70–80 bpm	fT4: 14.9 pmol/L NH_₃_: 39.8 *μ*mol/L	Discharged from hospital

1-month follow-up	Fully recovered, HR normal	No hyperammonemia detected	Continued management of hyperthyroidism

Abbreviations: CRRT, continuous renal replacement therapy; E, eye response; GCS, Glasgow Coma Scale; HR, heart rate (beat per minute); M, motor response; NH_₃_ serum ammonia; PTU, propylthiouracil; V, verbal response; V1t, verbal response score of 1 due to tracheal intubation.

^a^NA: not available at this timepoint.

^b^Prior treatment includes PTU (propylthiouracil), propranolol, Lugol's iodine, hydrocortisone, and antibiotics.

## Data Availability

The data that support the findings of this study are available from the corresponding author upon reasonable request.
